# Antimicrobial Properties of Bacterial Cellulose Films Enriched with Bioactive Herbal Extracts Obtained by Microwave-Assisted Extraction

**DOI:** 10.3390/polym14071435

**Published:** 2022-03-31

**Authors:** Ioana M. Bodea, Giorgiana M. Cătunescu, Carmen R. Pop, Nicodim I. Fiț, Adriana P. David, Mircea C. Dudescu, Andreea Stănilă, Ancuța M. Rotar, Florin I. Beteg

**Affiliations:** 1 Department of Preclinical and Clinical Sciences, Faculty of Veterinary Medicine, University of Agricultural Sciences and Veterinary Medicine Cluj-Napoca, 400372 Cluj-Napoca, Romania; ioana.bodea@usamvcluj.ro (I.M.B.); nfit@usamvcluj.ro (N.I.F.); florin.beteg@usamvcluj.ro (F.I.B.); 2 Department of Technical and Soil Sciences, Faculty of Agriculture, University of Agricultural Science and Veterinary Medicine Cluj-Napoca, 400372 Cluj-Napoca, Romania; 3 Department of Food Science, Faculty of Food Science and Technology, University of Agricultural Sciences and Veterinary Medicine Cluj-Napoca, 400372 Cluj-Napoca, Romania; carmen-rodica.pop@usamvcluj.ro (C.R.P.); andreea.stanila@usamvcluj.ro (A.S.); anca.rotar@usamvcluj.ro (A.M.R.); 4 Department of Mechanical Engineering, Technical University of Cluj-Napoca, 400114 Cluj-Napoca, Romania; mircea.dudescu@rezi.utcluj.ro

**Keywords:** lovage, oregano, rosemary, parsley, ethanol, microwave-assisted extraction

## Abstract

The use of bacterial cellulose (BC) as scaffold for active biofilms is one of the most interesting applications, especially for the biomedical and food industries. However, there are currently few studies evaluating the potential of incorporating herbal extracts into various biomaterials, including BC. Thus, the aim of this study is to report a screening of the total phenolic content and antioxidant and antimicrobial activity of ethanolic extracts of oregano, rosemary, parsley, and lovage. At the same time, the bioactive potential of BC enriched with the four ethanolic extracts is described. Microwave-assisted extraction was used to extract bioactive compounds from the four selected herbs. The physical, mechanical, structural, and chemical properties of BC were also assessed. Next, BC was enriched with the extracts, and their effect against *Escherichia coli*, *Staphylococcus aureus*, and *Candida albicans* was evaluated. The results showed that the bioactivity of the herbs varied significantly, with rosemary extract being the most bioactive. The BC films possessed good mechanical properties, and a three-dimensional network fibrillar structure appropriate for ethanolic-extract incorporation. The BC samples enriched with rosemary extracts had the highest antibacterial activity against *S. aureus*, while *E. coli*. and *C. albicans* seemed to be resistant to all extracts, regardless of herbs.

## 1. Introduction

In recent years, active materials have captured the focus of current research because of their properties that make them suitable for a wide variety of potential biomedical applications and uses in the food and paper industries [1,2]. The most sought-after properties are antimicrobial and antioxidant activities because they grant the material the bioactivity necessary for such applications. In general, an active material contains a scaffold that is enriched with an active substance [3].

Bacterial cellulose (BC) is a versatile structural material that can be shaped to accommodate for different uses [1,3]. It has been extensively used because of its hydrophilic nature, flexibility, nontoxicity, biocompatibility, aesthetic appearance, and good mechanical and barrier properties, which grant its wide availability [2,4]. However, BC itself has no antimicrobial and antioxidant activity [5]; therefore, to increase its applicability, BC can be enriched with some antimicrobial and antioxidant agents [3]. Some studies report the use of BC films enriched with different ethanolic extracts, such as mangosteen peel (*Garcinia mangostana*) [6], fireweed (*Epilobium angustifolium* L.) [7], bush guarri (*Euclea schimperi*) [8], and rosemary (*Rosmarinus officinalis*) extracts [3].

Because of the chaotic use of antibiotics, the occurrence of drug-resistant pathogens is increasing rapidly [9,10,11]. In this context, there is a constant need for new sources of antimicrobial agents [9,12,13]. This persistent problem has led to the constant exploration for new plant species with possible medicinal, antibacterial, and antioxidant properties [9,12,13]. Plants produce an extended range of bioactive molecules, being a rich source for different types of active substances. Thus, the continuous effort to find new phytochemicals with antibacterial potential against multiresistant bacteria has increased significantly [13]. Plant phenolic compounds belong to a major class of bioactive components and metabolites with bioactive potential attributed to antioxidant and antibacterial activities [14,15]. Free phenolic acids, present in ester or ether forms, are found in varying quantities in plant tissues. Different plant parts could, therefore, offer bioactive substances for food preservation and herbal medicine [15]. The antioxidant and antibacterial activity of herbs has been demonstrated in many studies over recent years [14,16,17,18,19].

Among other herbs, oregano (*Origanum vulgare*) and rosemary (*Rosmarinus officinalis*) (*Lamiaceae*), as well as parsley (*Petroselinum crispum*) and lovage (*Levisticum officinale*) (*Apiaceae*) are known for their antioxidant and antibacterial activity [16,17,18,19]. The bioactive-compound distribution during the development of leaves, flowers, stems, and roots and their biosynthetic pathways were previously studied [20]. In addition, the phytochemical profile and the correlations with their antioxidant and specific antimicrobial activity were also described [20,21]. Rosemary [22], oregano [23,24], parsley [15,25], and lovage [11,26] are described in the literature as valuable sources of phenolic compounds, phenolic acids, and flavonoids. Oregano and rosemary are rich in carvacrol and carnosic acid, which are responsible for their antimicrobial activity [10], while some isolated phenolics possess antiviral activity [27]. Meanwhile, parsley is rich in polyphenols and has remarkable antioxidant, antibacterial, and antifungal activities [25], and lovage is known for compounds with documented bioactive properties that are beneficial for human health [26].

The extraction method plays a crucial role in the bioactivity of the obtained compounds. Several methods are used to extract bioactive compounds from herbs belonging to the *Lamiaceae* and *Apiaceae* families, such as maceration [28], leaching [29], extraction with supercritical fluids (CO_2_) [30], dispersive liquid–liquid [29], sonication [31], enzymatic extraction [29], microextraction [29], and microwave-assisted extraction (MAE) [32,33,34]. However, conventional techniques of extracting active compounds are time- and solvent-consuming, thermally unsafe, and the analysis of plant constituents is limited by the extraction step [29]. The microwave-assisted extraction (MAE) technique offers some attractive features, such as high and fast extraction performance with less solvent consumption, extracting higher yields of bioactive compounds and offering protection to thermolabile constituents [33,35]. Over the past years, a large number of studies on microwave-assisted extraction have been made and remarkable results have been achieved. However, there are still many theoretical and technical hypotheses in the area of MAE that need to be overcome [36,37,38]. MAE of plant active compounds can be affected by a large variety of factors, such as concentration and type of solvent, microwave power, duration of microwave extraction, granulometry of plant samples, extraction temperature, and number of extraction cycles [36,38,39,40]. MAE is one of the most important techniques for extracting valuable compounds from plants, and it is quite adaptable on both a laboratory and industrial scale [36,38]. Recent trends in extraction technologies have focused on finding efficient and innovative procedures to acquire natural bioactive compounds, which could minimize extraction time, solvent consumption, and maximize yield recovery [38,39].

Although many studies have evaluated the effect of these parameters on the phenolic content and antioxidant activity of ethanolic extracts [41,42,43], to the best of our knowledge, there are no previous studies that assessed the influence of microwave-assisted extraction parameters on the antimicrobial activity of herbal extracts against the tested microbial strains. Therefore, we assume that this is the first study that intends to assess the influence of microwave-extraction parameters over the antimicrobial activity of rosemary, oregano, parsley, and lovage ethanolic extracts. Additionally, although various essential oils and herbal extracts together with their active components have been studied, there are very few studies assessing their activity when incorporated into various biomaterials, including cellulose [3,6,7,8]. To the best of our knowledge, only one other study has investigated the bioactivity of a material containing BC as scaffold and ethanolic extracts of herbs from the *Lamiaceae* and *Apiaceae* families [3]. Moreover, no current study has tackled the effect of various MAE parameters upon the chemical profile and potential bioactivity of BC enriched with parsley, lovage, rosemary, and oregano ethanolic extracts.

Thus, the aim of the present study is to assess the bioactivity of a material containing bacterial cellulose (BC) as a scaffold and ethanolic extracts of herbs from the *Lamiaceae* and *Apiaceae* families as active components. The obtained polymer is intended to be used as a bioactive material for food applications (active packaging) and biomedical uses (wound dressing). Natural extracts were proposed as active substances, which were extracted by using a green, environmentally friendly extraction procedure. Thus, MAE was used to extract bioactive compounds from rosemary, oregano, lovage, and parsley, and the total phenolic content and antioxidant and antimicrobial activity were evaluated. Additionally, the effect of various extraction parameters (ethanolic concentration, microwave power, extraction time, and repetition) were assessed upon the chemical profile and potential bioactivity of the extracts.

## 2. Materials and Methods

### 2.1. Microbial Strain and Chemicals

Microbial strain: *Gluconacetobacter xylinus* (*Komagataeibacter xylinus*) ATCC^®^ 700178™; *Escherichia coli* ATCC 25922; *Staphylococcus aureus* ATCC 6538P; *Candida albicans* ATCC 90028.

Chemicals: glucose (D-(+)-glucose anhydrous, Himedia, Mumbai, India); yeast extract (Himedia, Mumbai, India); CaCO_3_ (calcium carbonate, A.R., Himedia, Mumbai, India); Agar (Himedia, Mumbai, India); NaOH; NaOCl; 1.6% glutaraldehyde; sodium cacodylate trihydrate (C_2_H_12_AsNaO_5_) buffer; osmium tetraoxide 1%, uranyl acetate 2%; absolute ethanol (99.5%, *v*/*v*); HCl; gallic acid monohydrate; (±)-6-hydroxy-2,5,7,8-tetramethyl-chromane-2-carboxylic acid (Trolox); filter paper (Vtr lass s.r.o., PN/80 G/M2, pore size 8–11 μm); 2,2′-diphenyl-1-picrylhydrazyl radical (DPPH); methanol (Honeywell, Charlotte, North Carolina, United States); amoxicillin/clavulanic acid (20/10 µg/disc) (Himedia, Mumbai, India); miconazole 10 µg; nutrient broth (Biolab, Budapest, Hungary); glucose solution 10% (Hemopharm Beogradski, Vršac, Serbia); gallic acid monohydrate (≥98.0%).

### 2.2. Herb Samples and Ethanolic Extraction

Two herbs from the *Apiaceae* family (parsley (*Petroselinum crispum*) and lovage (*Levisticum officinale*)) and two from the *Lamiaceae* family (rosemary (*Rosmarinus officinalis*) and oregano (*Origanum vulgare*)) were used for the extraction. Dry parsley, lovage, rosemary, and oregano were purchased from a local producer. The microwave-assisted extraction (MAE) was performed using a microwave oven (Bluesky BMG20S-10). Ethanol was used as solvent because of its nontoxic nature and its relative common use [44]. Five grams of each plant were accurately weighed using an analytical balance, ground, placed in a 40 mL aqueous ethanol solution (40% *v*/*v*, 60% *v*/*v*, 80% *v*/*v*), and acidified with HCl (0.01% *v*/*v*) resulting in herbal ethanolic extracts with a concentration of 125 mg/mL dry weight (DW). The mixture was microwave-extracted using different microwave powers (160 W, 480 W, and 800 W) for different extraction periods to keep the solvent temperature below 40 °C. Preliminary extraction assays were performed varying the extraction time for each ethanol concentration, and the results were 10 s duration for 160 W and 800 W, and 40 s for 480 W. The heated mixtures were immediately cooled down to room temperature within 30 s using iced water (0–4 °C). The mixture was later repeatedly extracted up to 10 times, according to the protocol in Table 1. After filtration with filter paper (pore size 8–11 μm) the volume of extract solutions was adjusted to 40 mL with the same ethanol concentration as the extraction solvent [40].

The extraction conditions of MAE were selected according to previous literature results: a 1:8 herb:solvent ratio (*w*/*v*) was chosen because usually it can range between 1:5 [45] and 1:10 [46]. A maximum of 80% ethanolic concentration was chosen because although the reported ethanol concentration varied from 20% [47] to 100% [40], the usual maximum ranged between 75–80% in MAE [39,48,49], supporting our choice. The maximum microwave power of 800 W was chosen, because higher values decreased the extraction of bioactive components [39,48]. In addition, the reported microwave power varied from 140 W [40] to 900 W [37,48]. Previous reports also showed that repeated extraction steps gave the highest values of active compounds [40,50]. Thus, the extraction-repetition steps varied from 1–3 times [50,51], up to 10 times [40]. In this study, the extraction time for each ethanol concentration was of 10 s duration for 160 W and 800 W, and 40 s for 480 W. This was assessed after preliminary extraction assays, which ensured a solvent temperature below 40 °C to avoid thermal degradation of the active components [52].

### 2.3. Total Phenolic Content (TPC)

TPC in ethanolic extracts was determined spectrophotometrically following Folin–Ciocalteu method with absorbance in the Vis domain at a wavelength λ of 750 nm [15,42,53]. TPC was expressed in relation to a calibration curve with Gallic acid of different concentrations: 1 mg/100 mL; 0.5 mg/100 mL; 0.25 mg/100 mL; 0.125 mg/mL, and 0.0625 mg/mL. To plot the calibration curve, the absorbances were read according to Gallic acid concentrations. The calibration curve was: y = 0.9443x + 0.0608, having R^2^ = 0.9945.

TPC in the ethanolic extracts was determined using plates with 24 wells of 3 mL Each well contained 2.35 mL of distilled water; 0.05 mL of ethanolic extracts; 0.15 mL of Folin–Ciocalteu reagent; and 0.45 mL Na_2_CO_3_ (7.5%). The extracts were replaced with 0.05 mL of methanol for blanks. The samples were left in the dark for 2 h, and then the absorbance was measured at a wavelength λ of 750 nm (Biotek multidetector UV-Vis spectrometer) [25]. The total quantity of polyphenols was expressed in mg Gallic acid equivalents (GAE)/100 g dry weight (DW) using the calibration curve.

### 2.4. Antioxidant Activity, DPPH Assay

A modified version of 2,2-diphenyl-1-picrylhydrazyl (DPPH) method based on measuring the antioxidant-complexing ability of the stable radical DPPH was used to assess the antioxidant activity of the herbal extracts [16,25,42,54]. The reaction between DPPH and the antioxidants in the plant extracts was monitored at a wavelength λ of 515 nm (Biotek multidetector UV-Vis spectrometer). A methanol solution was used as blank; then, 1750 μL DPPH and 250 μL of sample were used for each determination. The absorbance was measured at 515 nm after 30 min [55]. The calibration curve was performed with Trolox using various dilutions (0.5 mM/L, 0.25 Mm/L, 0.125 Mm/L, 0.00915 mM/L) and then the absorbance was recorded for the studied samples. The obtained calibration curve had the following equation: y = 0.0029x + 0.0108 and R^2^ = 0.9985.

A stock methanolic DPPH solution was prepared by dissolving 80 µM DPPH in 98% methanol. The stock DPPH solution was freshly prepared, sonicated for 15 min, and stored in the dark at room temperature. A volume of 250 µL of sample was pipetted in a cuvette containing 1750 µL DPPH solution. The blank sample contained 150 µL DPPH and 250 µL methanol. The absorbance was measured at 515 nm after 30 min (Biotek multidetector UV-Vis spectrometer).

The antioxidant activity (AA) was expressed in mM Trolox equivalents (TE)/100 g DW using the calibration curve.

The percentile radical-scavenging activity (I%) was computed as

(1) $$I\%=\frac{A_{\mathrm{blank}}-A_{\mathrm{sample}}}{A_{\mathrm{blank}}}\cdot 100,\;(\%)$$

where A_blank_ = absorbance of stock DPPH solution; A_sample_ = absorbance of sample.

### 2.5. Antimicrobial Activity of Herbal Extracts

#### 2.5.1. Preparation of Microbial Strains

One Gram-negative bacteria (*Escherichia coli* ATCC 25922), one Gram-positive (*Staphylococcus aureus* ATCC 6538P), and one yeast strain (*Candida albicans* 90028) were used for the antimicrobial-activity assays. Both bacterial strains were replicated in 45 mL sterile nutrient broth (Oxoid Ltd., Basingstoke, Hampshire, UK) at 37 °C for 24 h. *C. albicans* was grown at 30 °C for 24 h. The purity of the inoculum was confirmed by plating on appropriate selective media and microscopic examination of the Gram-stained smear (Optika microscope, B252, M.A.D; Apparecchiature Scientifiche, Milan, Italy). A loopful of inoculum was transferred by streaking onto a selective medium: BairdeParker agar base supplemented with Egg Yolk Tellurite Emulsion (Oxoid Ltd.) for *S. aureus*; TBX agar (Oxoid Ltd.) for *E. coli*; and YPD agar (Oxoid Ltd.) for *C. albicans*. Plates were incubated for 24 h at 30 °C for *C. albicans*, while 37 °C for *S. aureus* and *E. coli*. The microbial cultures were maintained at refrigeration temperatures as stock cultures, in order to obtain freshly cultured microbial suspensions. This suspension was later used to assess the antimicrobial activity by disc-diffusion method [25,56].

Several colonies of standard cultures cultivated on Mueller–Hinton agar (Oxoid Ltd., Basingstoke, Hampshire, UK) were transferred in sterile saline solution (8.5 g/L) and adjusted to match the turbidity of McFarland 0.5 standard (1.5 × 10^8^ CFU/mL) [57]. Then, a bacterial suspension of 1.5 × 10^6^ CFU/mL was prepared. A volume of 15 mL of the Mueller–Hinton agar (Sifin Diagnostics GmbH, Berlin, Germany) was poured into Petri dishes. After the solidification of the medium, 100 μL culture suspension (1.5 × 10^6^ CFU/mL) was dispersed over the entire surface of the Mueller–Hinton agar (Sifin Diagnostics GmbH, Berlin, Germany) and YPD agar (Oxoid Ltd.) plate using a Drigalski spatula.

#### 2.5.2. Disc-Diffusion Method

The sterile discs (10 mm diameter) were placed in the inoculated Petri dishes, and each was loaded with 40 μL of plant ethanolic extracts. Ethanol 40%, 60%, and 80% were used as negative controls for their respective extracts, while amoxicillin/clavulanic acid (20/10 µg/disc), and miconazole (10 µg/disc) were used as positive controls. *S. aureus* and *E. coli* plates were incubated at 37 °C for 24 h while *C. albicans* was incubated at 30 °C for 24 h. The antimicrobial activity of the tested ethanolic extracts was assessed by measuring the inhibition-zone diameters, in mm, with a digital caliper. Each experiment was conducted in triplicate.

### 2.6. Obtaining of Bacterial Cellulose (BC)

Bacterial cellulose (BC) was synthesized by *G. xylinus* ATCC^®^ 700178™. The static-fermentation technique under aerobic incubation was performed throughout the study. *G. xylinus* was statically cultured in liquid media containing 50 g/L glucose, 5 g/L yeast extract, 12.5 g/L CaCO_3_, and distilled water as solvent [58] at 26 °C for 72 h. The culture was preserved on solid medium at 4 °C and recultivated as previously described every 2 to 3 weeks [59]. The inoculum solution was prepared from the solid-culture plates by vortexing (MaxQ 2000) 7 to 9 bacterial colonies of the 7-day old *G. xylinus* culture for 5 min in a 9 mL sterile saline tube. The resulted bacterial-cell suspension was adjusted to 1.5 × 10^7^ cells/mL [60] using a spectrophotometer (Shimadzu UV-1900) at 600 nm absorbance and used as inoculum solution [59].

The fermentation experiments were performed in sterilized 120 mL square glass bottles containing 100 mL specific liquid medium. Each flask was inoculated using 5 mL of the inoculum at 26 °C for 16 days [59]. All samples were performed in triplicate.

#### Purification of Bacterial Cellulose (BC)

After incubation, the BC films were removed from the surface of the liquid-culture medium, washed repeatedly (cca. 3 times) with distilled water, then treated with 500 mL of 0.1 M NaOH solution for 1 h at 80 °C on a magnetic stirrer, to remove all bacteria cells for 2 h [59]. Subsequently, the BC films (6.5 mm × 6.5 mm) were washed again with distilled water and left for 24 h in a 3% NaOCl solution [59,61]. Afterwards, the BC was washed with distilled water until reaching a neutral pH. Finally, the purified BC was stored in distilled water at 4 °C until further analysis.

### 2.7. Mechanical Properties

The mechanical properties of wet BC samples were determined using a tensile-testing machine (Instron 3366 (10 kN) in a tensile mode. All measurements were carried out at room temperature (23 °C) and humidity in the range of 45–50%. Samples of 6.5/2 cm and 0.5 mm thick were loaded to failure with constant crosshead speed (2 or 4 mm/min) [59,62]. Five specimens were tested and the maximum load (N), tensile strength (MPa), elongation at break (%), Young’s modulus (MPa), and stiffness (kN/cm) were calculated. The results were reported as mean ± SD of 5 measurements.

### 2.8. Scanning Electron Microscopy (SEM)

Scanning electron microscopy was used to assess the structure of nanofiber structure of the purified BC. All samples were prepared prior to SEM analysis by treatment with 1.6% glutaraldehyde in a sodium cacodylate trihydrate (C_2_H_12_AsNaO_5_) buffer (0.1 M, pH 7.4) for 1 h. Afterwards, each sample was washed 3–5 times every 5–10 min with the C_2_H_12_AsNaO_5_ buffer, and then left in the buffer solution for 1 d. Subsequently, the samples were washed 3–5 times every 5–10 min with the C_2_H_12_AsNaO_5_ buffer, and then left in the buffer solution overnight. Afterwards, each sample was lyophilized in a Critical Point drier, then sprayed with Au and Pd (80:20 ratio) in a sputtering apparatus (Leica EM ACE600). All prepared samples were analyzed with ZEISS EVO electronic microscope [59,63,64]. Fiber-diameter measurements were performed with ImageJ 1.48 software. The diameters were analyzed in 5 different image fields per each sample, measuring the diameter of minimum 100 fibers [59,65].

### 2.9. Preparation of the Enriched BC Films

The purified BC films were cut into 8 mm discs using a sterile biopsy punch (Henry Schein^®^, Melville, NY, USA). The discs were then pressed with filter paper until nearly all the water in their structure was removed, resulting in drained BC discs [59]. Afterwards, 10 discs were placed in each test tube containing 1.5 mL of plant extract, and loaded with extract for 24 h [57]. The enriched BC films were stored in test tubes containing plant extracts at 4 °C until further analysis.

### 2.10. Fourier Transform Infrared Spectroscopy (FT-IR)

The herbal extracts and the enriched BC were subjected to FT-IR analysis using the Shimadzu IR Prestige -21 spectrophotometer with horizontal ATR (Attenuated Total reflectance) diamond accessory with a single reflection from PIKE, using ethanol as background [59,61,64,66]. The spectra were recorded on the wavelength range 600–3500 cm^−1^, at a resolution of 4 cm^−1^, and 16 scans for one spectrum [59,67]. The absorption bands characteristic of the different types of bonds and functional groups (expressed in cm^−1^) were identified. The primary data obtained were processed using IR solution Software Overview (Shimadzu) and OriginR 7SR1 Software (OriginLab Corporation, Northampton, MA, USA.

### 2.11. Antimicrobial Activity of Herbal Extracts of Bacterial Cellulose (BC) Enriched with Herbal Extracts

The antimicrobial activity of BC enriched with ethanolic extracts was assessed as previously described, by loading the 8 mm BC discs in the ethanolic extracts for 24 h. Then the loaded BC discs, with approximately 15 µL of 125 mg/mL extract, were placed in inoculated Petri dishes. BC discs were also loaded in the respective ethanolic solutions (40%, 60%, and 80%) and used as negative control. BC films dipped in amoxicillin/clavulanic acid solution (30 µg/mL) were used as positive controls.

### 2.12. Statistical Analysis

XLSTAT (version 2021.3.1.1163) statistical software was used to analyze the results. A one-way ANOVA (*p* < 0.05) was used to compare the effects of extraction parameters (ethanol concentration, employed microwave power, and repetition) upon the properties of the ethanolic extracts (TPC, antioxidant and antimicrobial activities) and the enriched BC biofilms (antimicrobial activity). Fisher pairwise comparisons (LSD, *p* = 0.05) were employed whenever ANOVA indicated significant differences among the samples. Additionally, Pearson correlation coefficients were calculated between the TPC and antioxidant and antimicrobial activities. Linear regression analysis was used to quantify the effect of extraction parameters upon the bioactivity of the extracts and enriched BC with a confidence interval of 95%, a tolerance of 0.0001, and best model selection was done by adjusted R^2^.

A Correlational Principal Component Analysis (PCA) was also performed on the results formatted in observations/variables table, Pearson correlation with a significance level of 5% was used and distance correlational biplots were obtained [25,59,68]. Two of the five identified factors (components) were selected: F1, which had an Eigenvalue of 3.52 and accounted for a variability of 60.24%; and F2, which had an Eigenvalue of 1.87 and accounted for a variability of 32.00%.

## 3. Results and Discussion

### 3.1. Total Phenolic Content (TPC) of Ethanolic Extracts

The TPC of the selected herbs varied significantly (*p* < 0.0001), and overall, the highest TPC was obtained for rosemary ethanolic extracts (4176.79 mg GAE/100 g DW), followed by oregano (3829.45 mg GAE/100 g DW) and lovage (2783.15 mg GAE/100 g DW), while the lowest was parsley (600.33 mg GAE/100 g DW), regardless of the extraction conditions (Table 2). Additionally, the TPC was significantly different (*p* < 0.0001) between the two botanical families, as overall, the tested herbs from *Laminaceae* family had higher TPC than those from the *Apiaceae* family.

When comparing the TPC among the tested herbs, oregano extracted with a 40% ethanolic solution had the highest TPC (4314.06 ± 81.33 mg GAE/100 g DW), followed by rosemary extracted with 60% ethanolic solution (4240.28 ± 44.77 mg GAE/100 g DW) and 40% (4059.91 ± 11 mg GAE/100 g DW). An ethanolic solution of 40% extracted the highest TPC for lovage (3015.25 ± 70.02 mg GAE/100 g DW) and parsley (747.73 ± 21.32 mg GAE/100 g DW). All plant extracts with 80% ethanolic concentrations had the lowest phenolic content in all tested plants, with parsley having the lowest TPC (452.90 ± 81.89 mg GAE/100 g DW), as seen in Table 2. The TPC was significantly influenced by the concentration of the ethanolic solution used as extraction solvent (b = −11.596; *p* < 0.0001), as resulted from the linear regression analysis. In this sense, the increase in ethanolic concentration determined a linear decrease in TPC for all studied plants. Rosemary extracted with 80% ethanol (R1) had the highest TPC when compared with O1 (b = −347.34; *p* = 0.003), L1 (b = −1393.65; *p* < 0.0001) and P1 (b = −3576.46; *p* < 0.0001). Oregano, lovage, and parsley extracted with 40% ethanolic concentration had a higher phenolic content than rosemary, for which the highest TPC was for 60% ethanol, as seen in Table 2.

Similar to our results, Ramamurthy and Kannan [69] reported that the solvent extraction significantly affected the phenolic content of rosemary extracts as the concentration of TPC decreased in the following order: methanol > ethanol > water, with methanol being the most efficient solvent. However efficient, methanol poses toxicity concerns, and whenever possible, it should be replaced. Thus, it was shown that aqueous ethanolic solutions used as extraction solvent facilitated polyphenol extraction from herbs [18]. Water swells the plant matrix, allowing ethanol to penetrate the solid matrices easier, causing a disruption in the bonding between the bioactive compounds and plant matrix, and facilitating a better mass transfer of the compounds [70]. Previous results showed that ethanol was a good solvent for extracting bioactive substances from plants, and it was reported to be the best solvent to extract the phenolic compounds from rosemary leaves [18].

These TPC results are difficult to compare with other reports because of the different extraction methods, different solvents used, and also because of the units of measure used for their reporting [21,30,71,72,73]. Additionally, the TPC varies even within the same herb plant, by variant, pedoclimatic and agronomic factors, and depending on the part of the plant chosen for extraction or time of harvest [20,71,74].

Similar to our results, Celiktas et al. [30] reported that TPC varied from 34.1 to 119 mg GAE/g DW for rosemary. Aljabri [21] obtained much higher results, (65.5 to 211 mg GAE/g DW), and concluded that water was the best extract solvent (211 mg GAE/g DW) followed by ethanol (201 mg GAE/g DW) and finally ethyl acetate (65.5 mg GAE/g DW).

Similar results were obtained by Yesil-Celiktas et al. [75], who analyzed methanolic extracts of rosemary dried leaves harvested from different locations in Turkey and showed that the total phenols varied from 70.3 to 147.3 mg GAE/g DW. On the other hand, Bunghez et al. [20] reported a smaller quantity of polyphenols from rosemary, in both cold (10.83 mg GAE/g DW) and hot (15.31 mg GAE/g DW) water extraction. In addition, Vallverdu-Queralt et al. [76] extracted up to 5.02 mg GAE/g DW polyphenols from rosemary, using a hydroalcoholic solvent.

Dorman et al. [72] determined a higher TPC for water-soluble extracts obtained from three oregano species, and results varied from 77.6 mg GAE/g DW in toka oregano to 93.9 mg GAE/g DW and 119.1 mg GAE/g DW in Turkish oregano and Syrian oregano, respectively. Compared to our results, Henning et al. [73] reported up to two times higher TPC in dried oregano leaves (88.5 mg GAE/g DW). Rababah et al. [77] extracted a significantly higher TPC from oregano using methanol than ethanol, and observed that the extraction temperature had a significant effect on the TPC. The TPC from methanolic extracts varied from 3566.5 mg GAE/100 g DW at 60 °C to 1559.7 mg GAE/100 g DW at 20 °C, while for ethanolic extracts, TPC varied from 3207.3 mg GAE/100 g DW at 60 °C to 1101.4 mg GAE/100 g DW at 20 °C. In our case, the highest concentration of polyphenols was extracted with 40% ethanol (4314.06 ± 81.33 mg GAE/100 g DW), which is slightly higher. Our results for oregano extracts obtained with 60% and 80% ethanolic solutions, 3960.82 ± 94.39 mg GAE/100 g DW and 3213.48 ± 55.13 mg GAE/100 g DW, respectively, are similar to those reported by Alshwaikh et al. [78]. Although we attempted to maintain the extraction temperature below 40 °C, it was possible during the extraction for the temperature to have risen slightly in certain places in the static extraction vessel, and therefore, the results are closer to those obtained at 60 °C than 20 °C. Chun et al. [79] extracted the total polyphenols from oregano with hot water and varying concentrations (10–95%) of ethanol. The authors concluded that the highest TPC was found in water and 60% ethanol extracts (35.43 mg GAE/g DW). In our case, 60% ethanol extracted a smaller TPC (3960.82 ± 94.39 mg GAE/100 g DW) than 40% ethanol (4314.06 ± 81.33 mg GAE/100 g DW). On the contrary, Bunghez et al. [20] reported a smaller TPC for oregano, in both cold (15.35 mg GAE/g DW) and hot (16.56 mg GAE/g DW) water extraction.

In comparison to our results, Słowianek and Leszczyńska [74] extracted up to two times fewer polyphenols (17.8 mg GAE/g DW) from lovage by using a methanolic extraction. Similar studies reported a TPC of 19.70 mg GAE/g DW from lovage extracts, using hot demineralized water as solvent [80]. Previous results are in accordance with our study and report that the TPC for lovage methanolic extracts is significantly higher than for parsley [81].

Similar to our results for 40% ethanolic parley extracts (747.73 ± 21.32 mg GAE/100 g DW), Henning et al. [73] reported a TPC of 7 mg GAE/g DW in dried parsley extracted with water. On the other hand, Farah et al. [42] reported for parsley ethanolic extracts a higher TPC of 0.92 g GAE/100 g DW. Słowianek and Leszczyńska [74] extracted more polyphenols from parsley using methanol as solvent (13.6 mg GAE/g DW) compared to our results, which varied from 747.73 ± 21.32 mg GAE/100 g to 452.90 ± 81.89 mg GAE/100 g DW. Additionally, previous results are in accordance with our study and report that the TPC of oregano methanolic extracts is higher than lovage, which is in turn higher than parsley [74].

Pearson correlation analysis showed a correlation between the TPC of all tested plants with all the used extraction parameters. Firstly, it was observed that the TPC of all tested plants was correlated with the solvent concentration; however, not all correlations went in the same direction. Thus, only for rosemary a positive correlation was observed (r = 0.731; *p* = 0.025), while for oregano (r = −0.970; *p* < 0.0001), lovage (r = −0.901; *p* = 0.001), and parsley (r = −0.936; *p* < 0.0001) a negative correlation was obtained. As seen in Table 2, in the case of rosemary extracts, as solvent concentration increased, a higher amount of TPC was extracted, unlike oregano, lovage, and parsley extracts, where the highest TPC was extracted with 40% ethanolic solution.

The ethanolic concentration was negatively correlated with the TPC of *Laminaceae* plants (r = −0.504; *p* = 0.033) compared to the *Apiaceae* family, in which no such correlation was observed.

The microwave power was found to be negatively correlated with the TPC of lovage extracts (r = −0.807; *p* = 0.009), without any influence on the other tested extracts. The extraction time was positively correlated with the TPC of oregano (r = 0.740; *p* = 0.023) and parsley extracts (r = 0.811; *p* = 0.008), unlike rosemary, which was negatively correlated (r = −0.869; *p* = 0.002). The TPC of rosemary extract was also in a negative correlation with the extraction repetition (r = −0.774; *p* = 0.014).

### 3.2. Antioxidant Activity (AA) of Ethanolic Herbal Extracts

The AA of the tested aromatic plants was significantly different (*p* < 0.0001) among the four tested herbs. The highest AA and radical scavenging activity (I%) were obtained from rosemary ethanolic extracts (76.22 mM TE/100 g DW; 57.69%), followed by oregano (60.35 mM TE/100 g DW; 47.81%) and lovage (27.24 mM TE/100 g DW; 19.02%), while parsley had the lowest activity (7.60 mM TE/100 g DW; 5.51%) (Table 2). When comparing the antioxidant activity among the tested plants, the results showed that rosemary extracted with 60% and 80% ethanolic solutions had the highest activity of 87.23 ± 1.38 mM TE/100 g DW and 82.81 ± 0.83 mM TE/100 g DW, respectively. Similar results were obtained for I% with 65.01% and 62.04%, respectively. Oregano extracted with 40% ethanolic solution had the highest antioxidant activity (73.99 ± 1.66 mM TE/100 g DW; 57.05%) among the tested oregano samples, but lower than rosemary. Lovage and parsley showed the highest antioxidant activity for the 40% ethanolic extracts (32.06 ± 1.20 mM TE/100 g DW and 8.42 ± 1.11 mM TE/100 g DW, respectively). Similar results were observed for I% in both lovage and parsley extracted with 40% ethanol with 22.08% to 6.20%, respectively.

As in the case of TPC, comparison of the obtained results with the reported AA is difficult [80,82], because although there are many published studies, the results are significantly influenced by the extraction method and the analytical method used for the determinations. Additionally, the antioxidant compounds that are extracted depend on the solvent polarity, and most authors analyzed concentrations of plants similar to the portions used in common culinary practice [80]. Different solvents have been used in studies on the antioxidant capacity of plants, including acetone, methanol, ethanol, and water. This may explain the different results obtained for the same plant extracts [74].

Rosemary exhibited the highest AA, higher than oregano, lovage, and parsley. Ciolacu et al. [17] showed that rosemary acetone extracts had the highest AA, followed by oregano and parsley. On the contrary, Chrpova et al. [80] reported that among other tested culinary spices, oregano had the highest AA, almost 4 times higher than that of rosemary, with lovage having the lowest. Vallverdu-Queralt et al. [76] analyzed the antioxidant activity of rosemary ethanolic extracts and reported 1.98 mMol TE/g DW, which is more than twice compared to our highest obtained value (87.23 ± 1.38 mM TE/100 g DW).

Oregano exhibited a moderate antioxidant activity, lower than rosemary. Similar to our results obtained for 40% ethanolic oregano extract (73.99 ± 1.66 mM TE/100 g DW), Vallverdu-Queralt et al. [76] analyzed the antioxidant activity of oregano ethanolic extracts and reported an activity of 0.78 mM TE/g DW. Compared to our results, Wu et al. [83] reported an activity of 2001.29 µM TE/g DW using an acidified acetone water solvent, which is two-fold lower than our results obtained with 80% ethanol (48.61 ± 1.66 mM TE/100 g DW). A smaller AA (458.1 µM TE/g DW) was reported by Henning et al. [73] for water extracts. The authors also reported that oregano provided the strongest antioxidant activity compared, among others, to parsley [73]. Jałoszyński et al. [84] reported an AA of 168.87 µM TE/100 g DW in extracts with 80% aqueous methanol as solvent. Contrary to our results, Chrpova et al. [80] reported that oregano had the highest antioxidant capacity compared with rosemary, which had a medium activity. Gómez-Estaca et al. [85] reported that the results for antioxidant activity are higher for oregano water extract compared to rosemary extract.

Lovage exhibited a moderate AA, lower than rosemary and oregano, but higher than parsley. Similar to our reports, Nour et al. [81] recorded the highest AA in lovage, followed by celery, dill, and parsley. Słowianek and Leszczyńska [74] reported an AA of 41.4 µM TE/g DW in lovage ethanolic extracts.

Parsley exhibited a moderate AA compared to other tested plants. Similar to our results in all extracted ethanolic solutions of parsley, Wu et al. [83] reported an activity of 743.49 µM TE/g DW using an acidified acetone water solvent. Henning et al. [73] reported a smaller AA (59 µM TE/g DW) in water extracts. Parsley had an activity lower than rosemary and oregano, but much higher than lavender, paprika, and apple [17]. El-Zaeddi et al. [82] reported an activity of 16.27 mM TE/100 g DW from aqueous ethanolic extracts, which is twice higher than our results. In addition, El-Zaeddi et al. [82] showed that parsley had a higher AA than dill and coriander. Parsley had the highest ascorbic acid content compared with lovage, celery, and dill; however, it registered the lowest AA [81]. Furthermore, an AA of 16.8 µM TE/g DW [74] was recorded in parsley ethanolic extracts.

Overall, the results for the AA of ethanolic extracts followed a similar trend to TPC and are significantly different (*p* < 0.0001) for the two studied botanical families. The herbs from the *Laminaceae* family showed significantly higher AA than those from the *Apiaceae* family (Table 2). A similarly significant pattern (*p* < 0.0001) was observed for I%, with a value of 52.75% for the *Laminaceae* family and 12.26% for the *Apiaceae* family.

It was observed that the TPC was positively correlated with both AA and I% (r_AA_ = 0.921 and r_I%_ = 0.915; *p* < 0.0001) similar to previous reports [69,81,86].

Linear regression analysis showed that the AA was significantly influenced by the tested plant. Thus, rosemary extracts had the highest AA compared to parsley (b = −68.63; *p* < 0.0001), lovage (b = −48.99; *p* < 0.0001), and oregano (b = −15.85; *p* = 0.001). None of the other extraction parameters seemed to significantly influence the AA.

The TPC of the extracts was in direct relation with the AA (R^2^ = 0.848, *p* < 0.0001) and I% (R^2^ = 0.836, *p* < 0.0001), as resulted from linear regression analysis and seen in the obtained linear Equations (2) and (3), respectively.

AA = −9.16 + 0.18 TPC, (mM Trolox/100 g DW)(2)

I% = −7.57 + 0.14 TPC, (%)(3)

where AA—antioxidant activity; I%—percentile radical scavenging activity; TPC—total polyphenol content (mM TE/100 g DW)

Pearson correlation analysis showed that there are some correlations between the AA and I% of the tested extracts and extraction parameters. Thus, the ethanolic concentration was negatively correlated with the AA of oregano (r = −0.986; *p* < 0.0001), lovage (r = −0.898; *p* = 0.001), and parsley (r = −0.685; *p* = 0.042), but it was negatively correlated with AA of rosemary (r = 0.783; *p* = 0.013). Additionally, the extraction time was negatively correlated with rosemary AA (r = −0.869; *p* < 0.0001) and positively correlated with oregano (r = 0.916; *p* = 0.001) and lovage (r = 0.946; *p* < 0.0001) with no influence on parsley AA. The extraction repetition was positively correlated with AA of lovage (r = 0.741; *p* = 0.022), but negatively with AA from rosemary extracts (r = −0.925; *p* < 0.0001). The radical-scavenging activity of the tested plant extracts was also in direct correlation with the extraction parameters, in the case of rosemary and oregano. It was observed that the solvent concentration was positively correlated with rosemary AA (r = 0.779; *p* = 0.013) and negatively with that of the oregano extracts (r = −0.979; *p* < 0.0001). Moreover, a positive correlation was observed between oregano radical-scavenging activity and the extraction time (r = 0.940; *p* < 0.0001) and a negative correlation for rosemary (r = −0.983; *p* < 0.0001). The extraction repetition was only correlated with rosemary radical-scavenging activity (r = −0.924; *p* < 0.0001).

### 3.3. Antimicrobial Activity of Herbal Extracts

Rosemary extracted with 60% ethanol had the highest antibacterial activity against both *S. aureus* (4.50 ± 2.12 mm) and *E. coli* (4.50 ± 0.71 mm), but as expected, significantly lower than the control antibiotic (Table 3). Rosemary extracted with 80% ethanol had a similar activity against *S. aureus* (4.00 ± 0.00 mm), but lower against *E. coli* (3.50 ± 0.71 mm). This extract had the highest activity against *C. albicans* (2.50 ± 0.71 mm), compared to all the tested herbal extracts. Rosemary extracted with 60% ethanol had a lower activity against *C. albicans* (1.00 ± 0.00 mm). The 40% ethanolic extract had the lowest activity against *S. aureus* (2.00 ± 0.00 mm), while *E coli* and *C. albicans* showed resistance. Althobaiti [18] analyzed the effect of a 70% ethanolic extract against *S. aureus* and *E. coli* and observed that the extract displayed a high antibacterial activity against both Gram-negative and Gram-positive bacteria, similar with streptomycin used as control. Martinez et al. [87] evaluated the effect of rosemary aqueous extract against *S. aureus* and *E. coli* and showed an inhibition zone of 7.8 mm and 6.9 mm, respectively. The results are similar to our 60% ethanolic extracts (4.50 ± 2.12 mm and 4.50 ± 0.71 mm, respectively). Saǧdıç and Özcan [19] tested the activity of rosemary aqueous extract against *S. aureus* and *E. coli* and showed that it was ineffective. The antimicrobial activity of rosemary-leaf extract could be due to the presence of either phenolic compounds and flavonoids, but there is also evidence that minor chemical components (carnosol, rosmarinic acid, caffeic acid, diosmin, luteolin, zincquanine, camphor, cineole, and borneol) located in rosemary leaves have a significant effect on its antibacterial activity [18].

An 80% ethanolic oregano extract had the highest activity against *S. aureus* (1.50 ± 0.71 mm) and a lower activity against *E. coli* and *C. albicans* (1.00 ± 0.00 mm). The 60% ethanolic oregano extract had the highest antimicrobial activity against *E. coli* (3.00 ± 1.41 mm), compared to *S. aureus* and *C. albicans*, with an inhibition of only 1.00 ± 0.00 mm. In our study, a 40% ethanolic oregano extract only inhibited *S. aureus* (1.00 ± 0.00 mm), while *E. coli* and *C. albicans* were resistant (Table 3). Zazharskiy et al. [88] assessed the antibacterial effect of oregano ethanolic extract against *S. aureus*, and results showed a low activity (1.67 mm). In addition, oregano aqueous extracts showed high efficiency against *S. aureus*, with a higher antibacterial activity against *E. coli* [19]. However, Pasca et al. [89] stated that oregano ethanolic extracts had the highest antibacterial activity among the tested extracts against *E. coli* and *S. aureus* isolated from mastitis milk.

Lovage had a lower antibacterial activity compared to rosemary and oregano, but similar to that of parsley. The antibacterial activity against *S. aureus* of both 80% and 60% ethanolic extracts was similar to parsley 80% and 60% ethanolic extracts (1.50 ± 0.71 mm). All three tested microorganisms seemed to be resistant to 40% lovage extracts, while 40% parsley extract did slightly inhibit *S. aureus* (0.15 ± 0.07 mm). Ojala [90] reported that lovage methanolic extract (79 µg/mL) displayed a mild antimicrobial activity against *S. aureus. E. coli* and *C. albicans* were resistant to lovage extracts.

Zazharskiy et al. [88] reported that lovage ethanolic extract had a low antibacterial effect against *S. aureus* (2.27 mm). Previous studies reported that the antimicrobial activity of herbal extracts can be attributable to their content of phenolic compounds [42].

Among all tested parsley extracts, the 60% ethanolic extract had the highest antibacterial activity against *E. coli* (3.00 ± 1.41 mm) and *C. albicans* (2.00 ± 0.00 mm) compared to 80% extracts, which exhibited lower inhibition (1.50 ± 0.71 mm) against all three tested microorganisms (Table 3). Ojala [90] reported that parsley methanolic extract (37 µg/mL) displayed a low antibacterial activity against *S. aureus*, with no inhibitory activity against *E. coli* and *C. albicans*. Farah et al. [42] indicated that parsley-seed methanolic extracts displayed the highest antibacterial activity against *C. tropicalis*, with similar activity against *S. aureus*. A similar type of extract, but from parsley leaves, displayed a lower inhibitory activity [42]. Additionally, concentrations between 0.1 g/mL and 0.4 g/mL parsley 80% ethanolic extract showed no inhibitory activity against *S. aureus*; however, it was effective against *E. coli* (0.1 g/mL up to 0.4 g/mL) [91]. Aljabri [21] evaluated the antibacterial activity of cold and hot water extract from parsley, and results showed an inhibitory zone against *S. aureus* of 9.66 mm and 11 mm for 100 mg/mL and 150 mg/mL for cold water extracts, respectively, and 13.5 mm and 14.23 mm for hot water extracts of the same concentrations. Alshwaikh et al. [78] investigated the effect of parsley ethanolic and water extracts on bacteria isolated from urinary infections in children. Results showed that at a concentration of 12.5 of a 50 mg/mL, only the ethanolic extract had inhibitory activity on *E. coli* compared to the water extract, with no activity. However, both extracts had similar effect against *S. aureus*. Parsley ethanolic extract showed a low inhibitory activity on *S. aureus* (0.76 mm) [88].

Overall, of the four herbs, rosemary seemed to have the highest significant antimicrobial activity against the tested microorganisms. The highest activity was against *S. aureus*, and the lowest was against *C. albicans*. In accord with several previous studies, rosemary, oregano, lovage, and parsley had similar activities against all the tested microorganisms [21,42,78,88,89,90].

In terms of extraction parameters, it was observed that some were in correlation with the antibacterial activity against the tested bacterial strains. The ethanolic concentration was positively correlated with the inhibition of *S. aureus* (r = 0.403, *p* = 0.015), *E. coli* (r = 0.455; *p* = 0.005), and *C. albicans* (r = 0.762; *p* < 0.0001). This showed that a higher ethanolic concentration was able to extract bioactive compounds form the tested herbs with higher antimicrobial activity. Yang et al. [51] revealed that the extraction rate of polyphenols from *Phyllanthus emblica* improved when ethanol concentration increased. This could be because more liposoluble compounds were extracted over a high concentration of ethanol. In addition, the antibacterial activity of the extracts may be associated with their chemical composition and active compounds [41,42,43].

Although the microwave power was negatively correlated with the antimicrobial activity only in the case of *E. coli* inhibition (r = −0.358; *p* 0.032), it did show that the power employed during extraction is a factor worth studying. Alara et al. [48] indicated that the effect of the microwave power level was the major contributing variable, followed by ethanol concentration, when extracting a higher TPC yield from *Vernonia amygdalina* leaves. Microwave power was shown to significantly influence the concentration of metabolites from plant extracts [52]. Moreover, Alara et al. [39] observed that an increase in microwave power level caused a decrease in active compounds from *Vernonia amygdalina* and chokeberries (*Aronia melanocarpa*), respectively. Additionally, the extraction duration and repetitions were both negatively correlated with the inhibition of *S. aureus* (r_time_ = −0.465; *p* < 0.004; r_repetition_ =−0.403; p_repetition_ = 0.015); *E. coli* (r_time_ = −0.732, r_repetition_ = −0.813, *p* < 0.0001) and *Candida albicans* (r_time_ = −0.807 and r_repetition_ = −0.635, *p* < 0.0001) with the inhibition of the three tested microbial strains. This showed that the extraction parameters proposed for this study do influence the bioactivity of the extracts, and further studies are needed to assess and model their effect upon the extracts’ antimicrobial activity.

Overall, when comparing the antimicrobial activities exhibited by all the tested extracts, no statistically significant differences could be observed among the tested extraction method; thus, a general relationship could not be proposed.

However, as expected, differences were observed for each individual herb and tested microorganism. The linear regression analysis showed that the antibacterial activity against *S. aureus* was significantly influenced by the tested plant and by the extraction method (*p* < 0.0001, R^2^ = 0.821). Thus, rosemary extract had the highest antibacterial activity compared to oregano (b = −2.333; *p* < 0.0001), parsley (b = −2.450; *p* < 0.0001), and lovage (b = −2.500; *p* < 0.0001). Similar, Jalosinska and Wilczak [92] observed that rosemary ethanolic extracts possess a highly antibacterial power compared to lovage. Additionally, the influence of the ethanolic concentration on the antimicrobial activity of all tested herbs against *S. aureus* reached statistical significance (b = 0.045; *p* = <0.0001), showing that an increase in concentration caused an increase in antimicrobial activity. This could be because the extraction rate of active compounds, not necessarily phenols, seems to increase when ethanol concentration elevates [51], and thus increases the antibacterial activity, which is directly related to the active compounds [41,42,43]. Additionally, an increase of microwave power during extraction was shown to significantly decrease the efficiency of the extracts against *S. aureus* (b = −0.001; *p* = 0.005).

Rosemary seemed to have the highest activity against *E. coli*. The linear regression analysis showed that the antibacterial activity against *E. coli* significantly varied with tested plants, but also with the extraction method (*p* < 0.0001, R^2^ = 0.824). Rosemary extract had the highest antibacterial activity compared to lovage (b = −1.667; *p* < 0.0001), oregano (b = −1.333; *p* = 0.0001), and parsley (b = −1.167; *p* = 0.002). Additionally, similar to the results obtained for *S. aureus*, an increase in ethanolic concentration significantly increased the efficiency of the extracts against *E. coli* (b = 0.081; *p* < 0.0001), while an increase in microwave power caused a reduction in the antimicrobial activity (b = −0.005; *p* < 0.0001).

The antimicrobial activity against *C. albicans* showed that rosemary had the highest activity, followed by lovage and oregano (b = −0.500; *p* = 0.019). Parsley had no significant antimicrobial activity against *C. albicans*. In addition, similar with the two tested bacteria, the ethanolic concentration (b = 0.046; *p* < 0.0001) and microwave power (b = −0.001; *p* = 0.002) had a significant influence on the inhibition of *C. albicans*.

When analyzing the influence of TPC and AA on the antimicrobial activity of all tested herbal extracts against the tested microorganisms, it was observed that both parameters had a significant influence on the inhibition of the three tested microorganisms, with TPC having a very small negative effect (b = −0.001), and AA showing a positive influence (b*_S. aureus_* = 0.074, b*_E. coli_* = 0.069, b*_C. albicans_* = 0.031, *p* < 0.01). The present study shows that the proposed extraction parameters do influence the bioactivity of the extracts, and further studies are needed to assess their effect upon the extracts’ antimicrobial activity.

### 3.4. Principal Component Analysis of the Effect of Rosemary, Oregano, Lovage, and Parsley Extraction Parameters and Chemical Constituents on Selected Bacterial Strains

**Figure 1:**
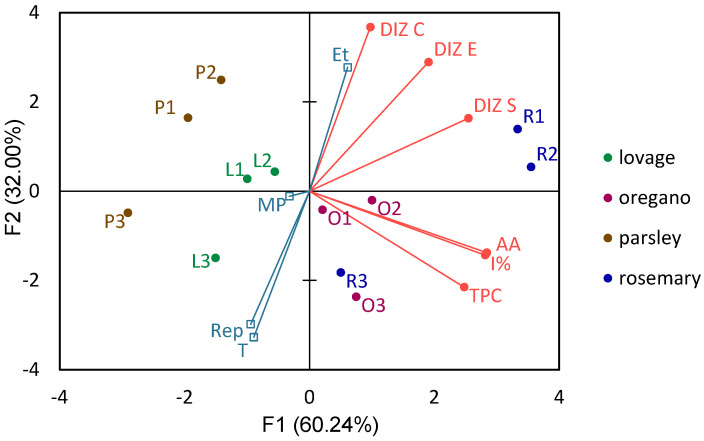
Principal component analysis (PCA) biplots of the samples and analyzed parameters of ethanolic extracts (axes F1 and F2: 92.24%), where R—rosemary; O—oregano; L—lovage; P—parsley; 1, 2, and 3—sample number according to the extraction procedure (Table 1); TPC—total phenolic content; AA—antioxidant activity; I%—radical-scavenging activity; Et—ethanolic concentration; MP—microwave power; Rep—extraction repetition, T—extraction duration.

The principal component analysis (PCA) (Figure 1, Table 4) shows the distribution of samples and the influence of extraction parameters (ethanol concentration, microwave power, extraction time, and repetition) on TPC and AA of the four tested plants (rosemary, oregano, lovage, and parsley). The two principal factors were analyzed, accounting for 92.24% of the total variability: F1, consisting of TPC, AA, I%, and *S. aureus* inhibition, and accounting for 60.24% of the variability in the samples; and F2, consisting of *E. coli* and *C. albicans* inhibition zone, with 32.00% of the variability.

It can be seen that a higher concentration of ethanol in the extraction solvent generated extracts with a higher antimicrobial activity, while the extraction time and repetitions seemed to negatively affect it.

The samples are grouped mainly by botanical family: *Apiaceae* herb is to the left of F1 origin, while *Lamiaceae* is to the right. This confirms the partial results presented on the TPC, AA, and inhibition of *S. aureus* where, overall, both rosemary and oregano showed higher bioactivity. An additional grouping can be seen by herb and extraction condition, as for all herbs’ extracts 1 and 2 were positioned very close together. This supports the partial results presented in the previous section where, generally, the extracts obtained with 60% and 80% and microwave powers of 160 W and 800 W, were extracted only once or repeated 5 times, respectively, and both were extracted for 10 s. The rosemary sample extracted in these conditions had the highest overall antimicrobial activity, while all oregano and R3 samples had the highest TPC content and antioxidant activity.

### 3.5. Characterization of the Physical Aspect of Bacterial-Cellulose (BC) Pellicles

**Figure 2:**
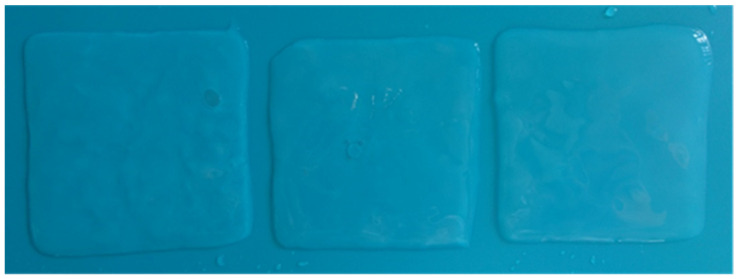
The aspect of the bacterial cellulose after purification.

**Figure 3:**
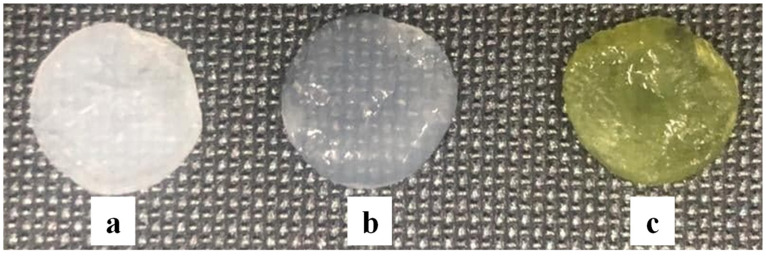
The aspect of bacterial cellulose (BC) film: (**a**) filter-paper-pressed film; (**b**) native purified BC film; (**c**) BC enriched with ethanolic herbal extract.

BC pellicles were produced in a static culture and harvested at the air–liquid interface of the culture medium. The native unpurified BC films were yellow, as reported previously [59]; thus, a purification treatment was necessary to attain transparency and to wash and eliminate all traces of bacteria and culture medium [61,64,93]. After the 0.1 M NaOH treatment, the pellicles became brownish, with random transparent areas similar to our previous results [59]. Following this purification, a 24 h 3% NaOCl treatment was employed [59] that made the BC films transparent, and the desired gel-like structure became visible (Figure 2 and Figure 3b).

The purification is a crucial step in the production of any cellulose product, and it is intended to remove all non-cellulose materials such as proteins and nucleic acids derived from bacterial cells and the culture medium. In our previous study [59], TEM imaging confirmed the effectiveness of the purification treatment, as no bacteria or other impurities were present in the internal structure of BC, even after 6 months. Thus, this treatment completely purified the BC films which ensured an up to 6-month storage, with no color and/or quality change, similar to our previous results [59].

The discs prepared for the absorption of natural extracts were pressed with filter paper until almost all the water in their structure was removed [57,59], resulting drained BC discs (Figure 3a). After a 24 h extract loading, the enriched BC absorbed acquired the natural green color of the herbal extracts (Figure 3c).

### 3.6. Mechanical Properties of Bacterial-Cellulose (BC) Pellicles

**Figure 4:**
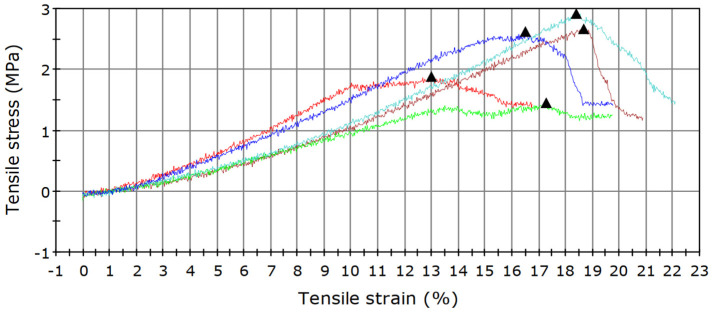
Tensile stress plotted by tensile strain of purified BC.

BC is an elastic malleable pellicle with increased strength and flexibility, which should maintain its integrity during use [94,95]. The mechanical testing (Figure 4) showed that wet BC films supported a maximum load of 2.77 ± 0.74 N and presented a tensile strength of 2.31 ± 0.61 MPa. The maximum load of pristine BC can reach up to 5.57 ± 0.38 N [59]. The tensile strength was in agreement with previous studies where it ranged between 0.75 ± 0.34 MPa [96] and 10.32 MPa [97]. The Young’s modulus and stiffness of BC were 13.83 ± 3.09 MPa and 2.76 ± 0.62 kN/cm, respectively. Our results are in line with current studies that reported a Young’s modulus ranging from 10.26 ± 0.35 MPa [96] to 26.38 ± 15.22 MPa [59] and a stiffness reaching values up to 5.28 ± 3.05 [59]. The elongation at break of BC was 16.75 ± 2.28 which is in accordance with other reports, and may vary between 5.49 ± 1.21% [96] up to 32.17% [95].

The mechanical properties of BC may vary dependent on culturing and processing methods. Process parameters such as culture time, medium, or post-treatment were shown to significantly influence maximum load, tensile strength, Young’s modulus, and elongation at break [59,95]. Additionally, the water in BC pellicles plays an active role in achieving a good alignment of the BC fibers. It limits the interactions of fibers and offers good mechanical properties because water can serve as a plasticizer [93].

### 3.7. Scanning Electron Microscopy (SEM) of Bacterial Cellulose (BC) Pellicles

**Figure 5:**
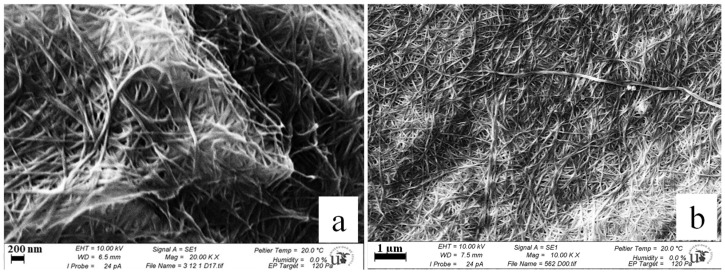
SEM images of surface morphology of purified BC. (**a**) aspect of BC fibrillar network structure; (**b**) aspect of BC surface morphology.

The structure of BC pellicles consisted of fibers that form an ultrafine network, with an average diameter ranging from 11 to 174 nm and a median of 48.14 ± 19.92 nm. This value is well in accord with our previous study, where values ranged between 40.60 ± 4.99 to 51.34 ± 6.99 nm [59]. Other authors reported an average diameter of 10–20 nm [98,99] up to 100–173 nm [96,99]. Figure 5a,b show a dense fibril 3D-matrix structure, which consists of randomly arranged nanofibers and empty spaces distributed randomly in between [95,100]. A 3D fibrillar network and a highly porous structure were observed, similar to previous reports [59,64,101,102]. This network with empty space in between helps the absorption and optimal release of water-soluble compounds from BC, with no structural effect upon the BC matrix [95,101]. Thus, BC is an attractive material for the fabrication of bioactive materials with delivery applications precisely because of its ultrafine fibrous network structure [103].

### 3.8. Fourier Transform Infrared Spectroscopy (FT-IR) of Bacterial-Cellulose (BC) Pellicles

**Figure 6:**
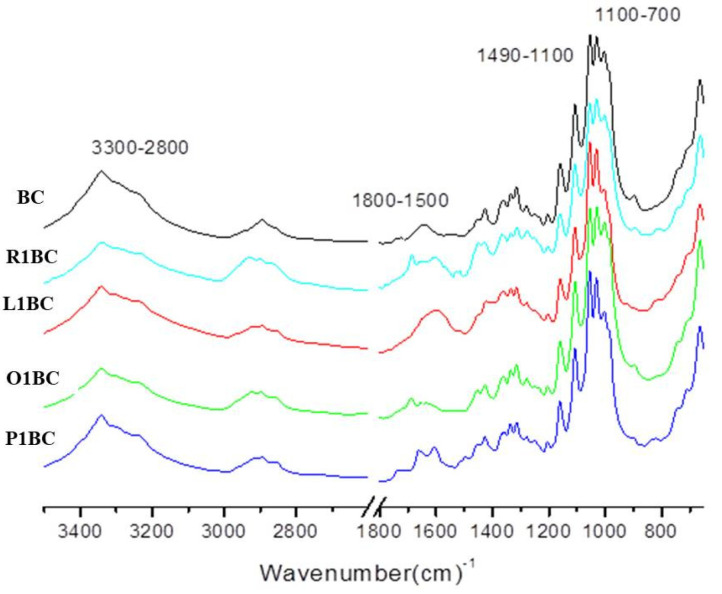
FT-IR spectra of bacterial cellulose (BC) and bacterial cellulose incorporated with rosemary extract (R1BC), lovage extract (L1BC), oregano extract (O1BC), and parsley extract (P1BC).

The Fourier Transform Infrared Spectrometry (FT-IR) technique is a nondestructive, fast, reproducible method for analyzing the intensities of vibration and rotation bands specific to molecules, because they differ in intensity and frequency [104]. The FT-IR technique highlights the functional groups that specifically absorb infrared (glycics, alcohols, aldehydes, ketones, acids). One of the methods usually employed to analyze BC is FT-IR because it analyzes BC using the chemical bonding present in the biopolymer. Therefore, the FT-IR method is an important alternative for qualitative analysis to identify the types and purity of BC [100,105]. The FT-IR spectrums of BC and BC, loaded with rosemary extract (R1BC), lovage extract (L1BC), oregano extract (O1BC), and parsley extract (P1BC), but also the phenolic-rich extracts alone (rosemary extract (R1), lovage extract (L1), oregano extract (O1), and parsley extract (P1)) were analyzed to obtain a comparative view on the functional groups in BC and plant extracts. Since BC was difficult to pulverize, the ATR mode, with 16 scans per measurement, between 400 cm^−1^ and 3500 cm^−1^, was used, according to previous studies [59,67,106]. Additionally, FT-IR was previously used to demonstrate the presence of phenolic compounds in biofilms and to provide evidence on how the incorporation of plant extracts alters the chemical structure of BC. The FT-IR spectra of the samples and the assignments of their infrared absorption bands are reported in Figure 6 and Table 5, respectively. The tentative assignments for bacterial cellulose (BC) were discussed in our previous study [59]. It can be seen that the presence of plant extracts does not induce many distinct spectral characteristics that could be directly assigned to the molecular structure of the extract components.

**Figure 7:**
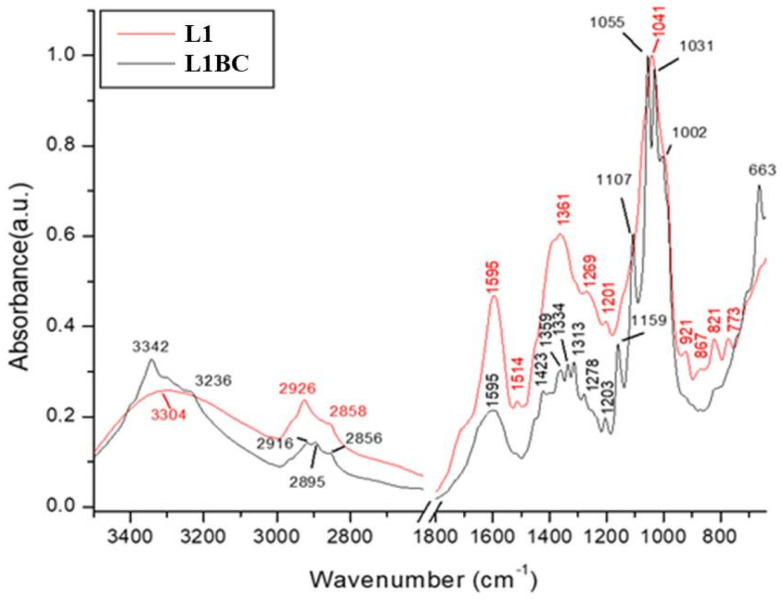
FT-IR spectra of lovage extract (L1) and bacterial cellulose incorporated with lovage extract (L1BC).

**Figure 8:**
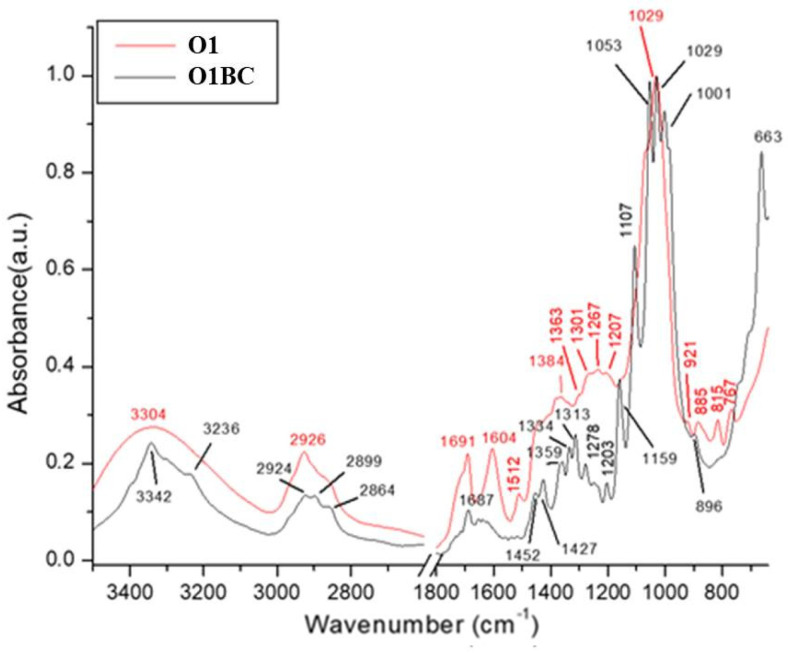
FT-IR spectra of oregano extract (O1) and bacterial cellulose incorporated with oregano extract (O1BC).

The adsorption band between 3000- and 3342 cm^−1^ present in all spectra could be attributed to stretching vibration of the intra- and inter-O–H bond in cellulose [107], or could be attributed to the associated OH-stretching vibrations (alcohols, phenols, and carboxylic acids) [108]. The absorption at the 3342 cm^−1^ peak was present in all BC samples, with or without extract loading (Figure 6, Figure 7 and Figure 8), but the absorbance peak was decreased to the lower wavenumber of 3338 cm^−1^ for the sample R1BC, probably due to the changing of the cellulose structure.

The FT-IR spectra of the herbal extracts (Figure 6, Figure 7 and Figure 8) showed a broad and intense band in the range of 3500–3100 cm^−1^ centered at 3307 cm^−1^ for R1, 3304 cm^−1^ for L1 and O1, and 3307 cm^−1^ for P1, which could be attributed to the associated OH-stretching vibrations (alcohols, phenols, and carboxylic acids) [108]. The absorption band between 2924 cm^−1^ and 2927 cm^−1^ for BC loaded with rosemary, lovage, oregano and parsley extracts, respectively all herbal extracts, could correspond to C–H stretching of the CH_2_ groups, CH_3_, and CH_2_ in aliphatic compounds, CH anti-sym and symmetric or CH_3_ attached to O or N, and could be an indicative of the chlorophyll groups [69,109,110].

The peaks at 2895 cm^−1^ for BC, R1BC, and L1BC, respectively 2899 cm^−1^ and 2894 cm^−1^ for O1BC and P1BC could be attributed to C–C stretching of CH_2_ and CH_3_ groups or CH_2_ asymmetric stretching [67]. For parsley extract and BC loaded with it, two peaks appeared at 1717 cm^−1^ and 1732 cm^−1^, respectively, which could be related to the presence of the stretching vibration of the polar group, attributed to the associated C=O of the carboxylic acid groups [111].

The bands in the region of 1700–1660 cm^−1^, present in all spectra, except for L1BC and L1, are characteristic of conjugate C=O and deconjugate C=O vibrations with aromatic or C=C stretching. In addition to the C=O-stretching vibration of quinones, conjugate carboxylic acid or ketones may overlap and absorb at the range of 1700–1630 cm^−1^ [108].

All FT-IR spectra of BC incorporated with herbal extracts presented peaks at 1604 cm^−1^ for R1BC, which decreased in the extract spectra to 1600 cm^−1^; 1595 cm^−1^ for L1BC and L1, 1600 cm^−1^ for O1BC, and 1604 cm^−1^ for O1; 1604 cm^−1^ for P1BC and 1606 cm^−1^ for P1, characteristic for stretching vibration of C–C [17]. The shifts in wavenumbers from the spectra of herbal extracts may be due to interactions with BC.

Weak peaks at 1514 cm^−1^ for R1 and L1, respectively 1512 cm^−1^ for O1 and P1, appeared only in the herbal extracts’ spectra, which could be attributed to the stretching vibration of C_ar_=C_ar_ in polar aromatic groups such as phenol [112]. The peaks at 1450 cm^−1^ and 1452 cm^−1^ present in all BC spectra incorporated with plant extracts could be probably due to the presence of the aromatic –C=C– bond [113]. The peak between 1423 cm^−1^ (for L1BC) and 1427 cm^−1^ (O1BC) present in all BC samples with or without plant extract may correspond to CH_2_ scissoring [114], but other studies assigned it to CH_2_ symmetric bonding or O–H in plane bending [115,116].

The peaks at 1359–1373 cm^−1^ present in spectra of all the enriched BC samples could be due to C–H bending of CH_3_ groups or to *ѵ* COO−antisymmetric stretching [117]. The peaks at 1334 cm^−^^1^ present in all BC samples may correspond to C–H deformation or O–H in-plane bending, and absorption at 1313 cm^−^^1^, also present in all BC samples, could be assigned to out-of-plane wagging of the CH_2_ groups [118].

All spectra, except for pure BC, present absorption bands between 1276 cm^−1^ and 1280 cm^−1^, which could be attributed to Ar-O in aryl ethers [119].

The absorption band at 1203 cm^−1^ present in all BC samples remained unidentified. As a typical indicator of the presence of C–O–C, antisymmetric bridge stretching of 1,4-b-O-glucoside in the absorption between 1157 cm^−1^ and 1159 cm^−1^ were observed in BC and all BC FT-IR spectra, shifting to a higher wavenumber (1160–1166 cm^−1^) for BC samples with plant extracts incorporated [103]. The weak absorption band at 1112 cm^−1^ for R1 and O1, respectively 1114 cm^−1^ for L1 and P1, could be attributed to the vibrations in alcohols (Csp3–OH), Csp3–OH of the carboxylic acids and Csp3–O of ester [120]. Castro et al. [118] reported that the peak at 1107 cm^−1^, present in all BC samples, indicated C–C bonds of the monomer units of polysaccharides or C–O-bending vibration.

It was shown that the peaks around 1000–1100 cm^−1^ could be assigned to C–O-stretching vibrations in primary alcohol and C–O–C skeletal vibrations [116], but some studies attributed the absorption at 1030 cm^−1^ and 1054 cm^−1^ to the bending of the C–O–H bond of the carbohydrate sun [121,122] or C–O–C pyranose-ring skeletal vibration [123,124], and the peaks around 1040 cm^−1^ could be due to carbohydrates of polysaccharides [125,126].

An intense peak placed at 1001 cm^−1^ and 1002 cm^−1^ was observed for all BC FT-IR spectra, originating from the stretching vibrations of C3–O3, which was the main bonding forming a cross-linking structure [127]. The 900–700 cm^−1^ band range corresponds to the out-of-plane deformation in substituted phenolic [128], polar compounds, and rocking of longue chains –(CH2)n– [112].

The weak absorbance band at 896 cm^−1^, 894 cm^−1^, 898 cm^−1^ present in all BC spectra was recorded and assigned to ν(C–O–C) in-plane, symmetric vibrations, characteristic for β-glycosidic bonds. Such bonds appear in cellulose with (1,4)-β-glycosidic linkages [129]. The peaks at 896 cm^−1^, 894 cm^−1^, 898 cm^−1^ present in all BC spectra could be assigned to antisymmetric out-of-phase ring stretching of beta-glycosidic linkages between the glucose units, which is designated as an amorphous absorption band [130,131]. The peaks at 661 cm^−1^, for L1 and O1; 663 cm^−1^ for R1 and P1, shifted to higher wavenumbers in samples of bacterial cellulose incorporated with these extracts, and could be assigned to C–OH in alcohols of C–O–H bending [119].

### 3.9. Antimicrobial Activity of Bacterial Cellulose (BC) Enriched with Herbal Extracts

Only one extract per herb was selected for the antimicrobial activity testing of BC, based on the results obtained in the antimicrobial activity assay (Table 3) and following the PCA (Figure 1). Thus, one common extraction protocol was chosen for the four herbs, which would produce extracts with the highest overall antimicrobial activity against the three tested microorganisms (80% ethanol, 800 W, 10 s, five repetitions).

As previously reported [3], pure BC showed no antimicrobial effect against the two tested bacterial strains, nor *Candida albicans*. All enriched BC biofilms inhibited *S. aureus*, except for parsley extracts (Table 6). Additionally, a significant difference was observed among the tested extracts; thus, rosemary had the highest antibacterial activity against *S. aureus* (5.15 ± 0.5 mm), followed by lovage (2.15 ± 0.5 mm) and oregano (1.15 ± 0.5 mm). However, *E. coli* and *C. albicans* seemed to be resistant to the activity of the extracts. The lower antimicrobial activity than that obtained for the extracts can be explained by the fact that the volume of extract loaded onto the 8 mm BC disc was lower than that loaded into the antibiogram disc (15 μL vs. 40 μL). Thus, BC might need to be loaded with a more concentrated extract to obtain the same efficiency. An additional factor that needs to be considered is that the natural fibrillar network of BC is different than that of antibiogram discs, which interferers with the drug-release properties. Drug-release activity from hydrogels can be influenced by many factors, such as drug concentration and characteristics, swelling, and the hydrogel structure [132]. Unlike traditional paper, BC is insoluble in water [3,133], and even though filter paper has higher inhibition zones compared to enriched BC, it should be taken into consideration that BC has higher mechanical strength compared to plant cellulose [133,134], as well as a unique fibrillar structure [135] higher liquid absorption and expansion capacity [136] so it is worth using this biomaterial in further research [133]. In addition, 3D fibrillar structure of BC can facilitate drug uptake and faster drug-release activity because of the larger surface area [136].

Similar to our study, Moradian et al. [3] showed that BC enriched with rosemary aqueous extracts (25% and 50% DW) exhibited a higher efficiency against *S. aureus* (20 and 25 mm, respectively) than *E. coli* (17 and 22 mm, respectively), with higher inhibition zones.

Santos et al. [1] incorporated oregano essential oil in BC film and assessed its activity against *E. coli* and *S. aureus*. The results showed that *E. coli* was more resistant than *S. aureus*, supporting our results. Other studies reported the use of BC films enriched with different ethanolic extracts, such as mangosteen peel (*Garcinia mangostana*) [6], fireweed (*Epilobium angustifolium* L.) [7], and bush guarri (*Euclea schimperi*) [8], but the antimicrobial activity was not assessed.

## 4. Conclusions

Both TPC and AA of the selected herbs varied significantly, with rosemary having the highest TPC and AA overall, followed by oregano, lovage, and parsley. In this sense, a significant linear relationship between TPC and AA was obtained. The four herbs showed significant antimicrobial activity, which varied by herb, extraction procedure, and tested microorganism. Rosemary extracted with 60% ethanol had the highest antimicrobial activity against both *S. aureus* and *E. coli*, while the extract obtained with 80% ethanol presented the highest activity against *C. albicans.* Lovage and parsley had a lower antimicrobial activity. All extraction parameters seemed to significantly influence the bioactivity of the extracts, with the solvent concentration having the most evident effect. Thus, results showed that the extraction parameters proposed for this study do influence the antimicrobial activity of the extracts, and further studies are needed to assess and model their effect upon the extracts’ bioactivity, since there are no other studies to prove it. Additionally, bacterial cellulose obtained in this study had a good transparency after the purification treatment and an optimal 3D network that allowed the loading of herbal extracts. The median cellulose-fiber diameter was of 48.14 ± 19.92 nm. Mechanical tests showed a maximum load of 2.77 ± 0.74 N and it presented a tensile strength of 2.31 ± 0.61 MPa.

Only one extract per herb, with the highest overall bioactivity, was selected for further testing of BC. Thus, one common extraction protocol (80% ethanol, 800 W, 10 s, 5 repetitions) was chosen for the four herbs. FT-IR was used to demonstrate that the presence of the extracts in BC structure did not induce many distinct spectral characteristics that could be directly assigned to the molecular structure of the extract components. The antimicrobial activity of enriched BC varied based on herb and target microorganism. Thus, rosemary extract had the highest antimicrobial activity against *S. aureus*, followed by lovage and oregano, while parsley extract seemed to have no significant effect. *E. coli* and *C. albicans* seemed to be resistant to all extracts, regardless of herbs.

Thus, the addition of herbal ethanolic extract to BC seems to be a promising technique in obtaining a biodegradable biofilm with proven antimicrobial properties granted by natural antimicrobials. However, these are only proof-of-concept preliminary results that set the stage for further studies that need to properly describe the drug-release dynamic of the enriched BC. Additionally, the extrapolation of in vitro results needs to be validated in situ for food-related applications, or in vivo for biomedical uses.

## Figures and Tables

**Table 1:** Extraction conditions of microwave-assisted extraction for parsley, lovage, rosemary, and oregano.

Sample	Ethanol Concentration (% *v*/*v*)	Microwave Power (W)	Extraction Time (s)	Extraction Repetitions
1	80	800	10	5
2	60	160	10	1
3	40	480	40	10

**Table 2:** Total polyphenol content and antioxidant activity of parsley, lovage, rosemary, and oregano by microwave-assisted extraction.

Botanical Family	Herb	Sample	Ethanol (%)	Microwave Power (W)	Extraction Time (s)	Times Extracted	TPC (mg GAE/100 g DW)	AA (mM TE/100 g DW)	I (%)
*Lamiaceae*	Oregano *Origanum vulgare*	O1	80	800	10	5	3213.48 ± 55.13 ^c^	48.61 ± 1.66 ^e^	40.39 ± 2.02 ^d^
		O2	60	160	10	1	3960.82 ± 94.39 ^b^	58.54 ± 0.96 ^d^	46.01 ± 1.18 ^c^
		O3	40	480	40	10	4314.06 ± 81.33 ^a^	73.99 ± 1.66 ^c^	57.05 ± 2.08 ^b^
	rosemary *Rosmarinus officinalis*	R1	80	800	10	5	4230.19 ± 88.30 ^a^	82.81 ± 0.83 ^b^	62.04 ± 1.13 ^a^
		R2	60	160	10	1	4240.28 ± 44.77 ^a^	87.23 ± 1.38 ^a^	65.01 ± 1.77 ^a^
		R3	40	480	40	10	4059.91 ± 11.20 ^b^	58.63 ± 1.57 ^d^	46.04 ± 1.94 ^c^
*Apiaceae*	lovage *Levisticum officinale*	L1	80	800	10	5	2385.02 ± 21.69 ^e^	24.15 ± 1.52 ^g^	16.90 ± 1.89 ^f^
		L2	60	160	10	1	2949.17 ± 40.35 ^d^	25.53 ± 0.97 ^g^	18.09 ± 1.20 ^ef^
		L3	40	480	40	10	3015.25 ± 70.02 ^d^	32.06 ± 1.20 ^f^	22.08 ± 1.50 ^e^
	parsley *Petroselinum crispum*	P1	80	800	10	5	452.90 ± 81.89 ^h^	6.58 ± 0.57 ^h^	4.57 ± 0.70 ^g^
		P2	60	160	10	1	600.35 ± 45.25 ^g^	7.78 ± 1.10 ^h^	5.77 ± 1.46 ^g^
		P3	40	480	40	10	747.73 ± 21.32 ^f^	8.42 ± 1.11 ^h^	6.20 ± 1.32 ^g^

Note: The data are presented as mean ± SD (*n* = 3). TPC = Total polyphenol content; AA = Antioxidant activity; I = percentile radical-scavenging activity; GAE-Gallic acid equivalents; TE = Trolox equivalents; Values with different letters (a–h) in the same column differ significantly (Fisher (LSD), *p* < 0.05).

**Table 3:** Antimicrobial activity of parsley, lovage, rosemary, and oregano extracts against *S. aureus*, *E. coli*, and *Candida albicans*, assessed by the diameter of inhibition zone (DIZ).

Botanical Family	Herb	Sample	Ethanol (%)	Microwave Power (W)	Extraction Time (s)	Extraction Repetition	DIZ *S. aureus* (mm)	DIZ *E. coli* (mm)	DIZ *C. albicans* (mm)
*Lamiaceae*	Oregano *Origanum vulgare*	O1	80	800	10	5	1.50 ± 0.71 ^cd^	1.00 ± 0.00 ^ef^	1.00 ± 0.00 ^d^
		O2	60	160	10	1	1.00 ± 0.00 ^cd^	3.00 ± 1.41 ^bcd^	1.00 ± 0.00 ^d^
		O3	40	480	40	10	1.00 ± 0.00 ^cd^	R ^f^	R ^e^
	rosemary *Rosmarinus officinalis*	R1	80	800	10	5	4.00 ± 0.00 ^b^	3.50 ± 0.71 ^bc^	2.50 ± 0.71 ^b^
		R2	60	160	10	1	4.50 ± 2.12 ^b^	4.50 ± 0.71 ^b^	1.00 ± 0.00 ^d^
		R3	40	480	40	10	2.00 ± 0.00 ^c^	R ^f^	R ^e^
*Apiaceae*	lovage *Levisticum officinale*	L1	80	800	10	5	1.50 ± 0.71 ^cd^	1.00 ± 0.00 ^ef^	1.00 ± 0.00 ^d^
		L2	60	160	10	1	1.50 ± 0.71 ^cd^	1.00 ± 0.00 ^cde^	1.00 ± 0.00 ^d^
		L3	40	480	40	10	R ^d^	R ^f^	R ^e^
	parsley *Petroselinum crispum*	P1	80	800	10	5	1.50 ± 0.71 ^cd^	1.50 ± 0.71 ^def^	1.50 ± 0.71 ^cd^
		P2	60	160	10	1	1.50 ± 0.71 ^cd^	3.00 ± 1.41 ^bcd^	2.00 ± 0.00 ^bc^
		P3	40	480	40	10	0.15 ± 0.07 ^d^	R ^f^	R ^e^
		amoxicillin/ clavulanic acid					18.50 ± 0.24 ^a^	7.00 ± 0.47 ^a^	NA
		miconazole					NA	NA	10.83 ± 0.24 ^a^

Note: The data are presented as mean ± SD (*n* = 3). DIZ—diameter of inhibition zone (mm); R—resistant; NA—not applicable. Values with different letters (a–f) in the same column differ significantly (Fisher (LSD), *p* < 0.05).

**Table 4:** Correlations between variables and principal component analysis (PCA) factors, contribution of the variables and squared cosines of the variables.

PCA Variable	Correlations between Variables and PCA Factors		Contribution of the Variables (%)		Correlations between Variables and PCA Factors	
	F1	F2	F1	F2	F1	F2
TPC	0.82	−0.52	18.50	13.94	**0.67**	0.27
AA	0.94	−0.33	24.31	5.71	**0.88**	0.11
I%	0.93	−0.34	23.87	6.19	**0.86**	0.12
DIZ S	0.84	0.39	19.51	8.06	**0.71**	0.15
DIZ E	0.63	0.70	10.95	25.28	0.40	**0.49**
DIZ C	0.32	0.89	2.87	40.83	0.10	**0.78**
Et	0.20	0.67			0.04	**0.45**
MP	−0.11	−0.03			0.01	0.00
T	−0.29	−0.79			0.09	**0.62**
Rep	−0.31	−0.72			0.10	**0.52**

Note: Values in bold correspond for each variable to the factor for which the squared cosine is the largest. TPC—total phenolic content; AA—antioxidant activity; I%—radical scavenging activity; Et—ethanolic concentration; MP—microwave power; Rep—extraction repetition, T—extraction duration.

**Table 5:** Tentative assignments of some functional groups on bacterial cellulose (BC), bacterial-cellulose pellicles enriched with ethanolic extracts of rosemary (R1BC), lovage (L1BC), oregano (O1BC), and parsley (P1BC) and ethanolic extracts of rosemary (R1), lovage (L1), oregano (O1), and parsley (P1) by FT-IR.

Samples Wavenumber (cm^−1^)									Tentative Assignments	References
BC	R1BC	R1	L1BC	L1	O1BC	O1	P1BC	P1	Functional Group	
	665	663	663	661	663	661	665	663	C–OH in alcohols of C–O–H bending	[119]
		767		773		767		770	γ C–Har (2C–Har adjacent) out-of-plane deformation of Aromatic groups	[112,128]
		815		821		816		820		
896	894		896		898		898		*ѵ s* (C–O–C) in-plane	[129,130]
1002	1001		1002		1001		1001		C–3…O–3 stretching	[127]
1029	1029	1031	1030	1031	1029	1029	1031	1033	Bending of C–O–H bond of carbohydrates	[121]
	1043		1041		1045		1041		*ѵ* C–O stretching of polysaccharides or polysaccharide-like substances	[125,126]
1055	1055		1055		1053		1055		C–O stretching vibrations in primary alcohol; The bending of C–O–H bond of carbohydrates or C–O–C pyranose ring skeletal vibration	[116,121,122,123,124]
1109	1107		1107		1107		1107		C–C bonds of the monomer units of polysaccharide or C–O bending vibration	[118]
		1112		1114		1112		1114	*ѵ* C–O stretching of aryl ethers and phenols	[120]
1157	1161	1161	1159	1160	1159	1163	1161	1166	C–O–C antisymmetric bridge stretching of 1,4-b-D-glucoside	[103]
1203	1203		1201		1207		1203		Unidentified	
	1276	1280	1278	1269	1278	1267	1278	1279	Ar–O in aryl ethers	[119]
1313	1313		1313		1313		1313		Out-of-plane wagging of the CH_2_ groups	[118]
1334	1336		1334		1334		1334		C–H deformation or O–H in-plane bending	[118]
	1373	1371	1359	1361	1363	1359	1369	1370	*ѵ* COO– antisymmetric stretching C–H bending of CH_2_ and CH_3_ groups	[117]
1427	1425		1423		1427		1425		CH_2_ symmetric bending or O–H in plane bending	[114,115,116]
	1450		1450		1452		1450		Aromatic –C=C– bond	[113]
		1514		1514		1512		1512	*ѵ* Car=Car aromatic-stretching vibrations	[112]
	1604	1600	1595	1595	1600	1604	1604	1606	Stretching vibration of C–C	[17]
-	1686	1697			1687	1691	1660		*ѵ* C=O stretching of amide groups (Amide I band), C=O of quinone and/or H–bonded conjugated ketones	[108]
-							1732	1717	*ѵ* C=O stretching vibrations in associated carboxyl COOH, ketone groups, and esters	[111]
2895	2895		2895		2899		2894		C–H stretching of CH_2_ and CH_3_ groups	[67]
-	2926	2927	2925	2926	2924	2926	2924	2926	CH_3_ and CH_2_ in aliphatic compounds, CH anti sym and symmetric or CH_3_ attached to O or N	[69,109,110]
3342	3342	3307	3342	3304	3342	3304	3342	3307	O–H stretching vibration or *ѵ* (OH) hydroxyl groups in (phenols, alcohols, and organic acids)	[107,108]

Note: BC—bacterial cellulose, R1—rosemary ethanolic extract, P1—parsley ethanolic extract, O1—oregano ethanolic extract, L1—lovage ethanolic extract, R1BC—BC pellicles enriched with rosemary ethanolic extract, L1BC—BC pellicles enriched with lovage ethanolic extract, O1BC—BC pellicles enriched with oregano ethanolic extract, P1BC—BC pellicles enriched with parsley ethanolic extract.

**Table 6:** Antimicrobial activity of bacterial cellulose enriched with parsley, lovage, rosemary, and oregano extracts against *S. aureus*, *E. coli*, and *Candida albicans*.

Sample	DIZ *S. aureus* (mm)	DIZ *E coli* (mm)	DIZ *C. albicans* (mm)
O1	1.15 ± 0.5 ^b^	R	R
R1	5.15 ± 0.5 ^a^	R	R
L1	2.15 ± 0.5 ^b^	R	R
P1	R	R	R
BC	R	R	R
amoxicillin/ clavulanic acid	19.5 ± 0.5	R	NA
miconazole	NA	NA	7.5 ± 0.5

Note: The data are presented as mean ± SD (*n* = 3). DIZ—diameter of inhibition zone (mm), R—resistant; NA—not applicable. Values with different letters (a, b) in the same column differ significantly (Fisher (LSD), *p* < 0.05).
